# Reproductive fluids, used for the *in vitro* production of pig embryos, result in healthy offspring and avoid aberrant placental expression of *PEG3* and *LUM*

**DOI:** 10.1186/s40104-020-00544-0

**Published:** 2021-02-15

**Authors:** E. París-Oller, S. Navarro-Serna, C. Soriano-Úbeda, J. S. Lopes, C. Matás, S. Ruiz, R. Latorre, O. López-Albors, R. Romar, S. Cánovas, P. Coy

**Affiliations:** 1 Department of Physiology- Faculty of Veterinary, University of Murcia - Campus Mare Nostrum, 30100 Murcia, Spain; 2 Institute for Biomedical Research of Murcia, IMIB-Arrixaca, Murcia, Spain; 3 Department of Anatomy and Comparartive Pathology, Faculty of Veterinary Medicine, University of Murcia - Campus Mare Nostrum, 30100 Murcia, Spain

**Keywords:** Assisted reproductive technologies, Embryo transfer, *In vitro* embryo production, Placenta, Reproductive fluids

## Abstract

**Background:**

*In vitro* embryo production (IVP) and embryo transfer (ET) are two very common assisted reproductive technologies (ART) in human and cattle. However, in pig, the combination of either procedures, or even their use separately, is still considered suboptimal due to the low efficiency of IVP plus the difficulty of performing ET in the long and contorted uterus of the sow. In addition, the potential impact of these two ART on the health of the offspring is unknown. We investigated here if the use of a modified IVP system, with natural reproductive fluids (RF) as supplements to the culture media, combined with a minimally invasive surgery to perform ET, affects the output of the own IVP system as well as the reproductive performance of the mother and placental molecular traits.

**Results:**

The blastocyst rates obtained by both *in vitro* systems, conventional (C-IVP) and modified (RF-IVP), were similar. Pregnancy and farrowing rates were also similar. However, when compared to *in vivo* control (artificial insemination, AI), litter sizes of both IVP groups were lower, while placental efficiency was higher in AI than in RF-IVP. Gene expression studies revealed aberrant expression levels for *PEG3* and *LUM* in placental tissue for C-IVP group when compared to AI, but not for RF-IVP group.

**Conclusions:**

The use of reproductive fluids as additives for the culture media in pig IVP does not improve reproductive performance of recipient mothers but could mitigate the impact of artificial procedures in the offspring.

**Supplementary Information:**

The online version contains supplementary material available at 10.1186/s40104-020-00544-0.

## Background

In last decades, Assisted Reproductive Technologies (ART) have become more relevant, not only in the human species, but also in animal production. Artificial insemination (AI) in pigs and embryo transfer (ET) in cows are nowadays common practices for animal production in commercial farms [1, 2]. Due to the continuous growth of the world population, there is an interest in improving production strategies and reducing costs in farming for food. To reach these goals, some ART are envisioned as potential tools [3–5].

According to the latest report published by the Trade and Markets Division of FAO [6], pork is one of the most consumed meat in the world, and the prediction is that, particularly in some Asian countries, their consumption and demand will continue to increase [7]. To respond to such demands, there is a growing necessity to develop new technologies to produce pig embryos from gametes of high genetic value at large scale [4]. However, the use of *in vitro* produced embryos (IVP) to increase meat production is still far from being widely established in the pig.

Indeed, according to the International Embryo Technology Society (IETS) in 2018 [1], there was no recorded use of porcine embryos for embryo transfers. In 2017, this society reported that 43 fresh *in vivo*-derived embryos were transferred. In 2016, only 397 pig embryos were collected worldwide *in vivo* and none of them were produced *in vitro* [8]. Although not all centres/institutions report their information, it is evident that porcine embryo collection is almost non-existing compared to the bovine species, where 935,000 embryos were obtained *in vivo* in 2016 and 2600 were produced *in vitro* from oocytes obtained from ovaries collected at slaughterhouses [8].

One of the most consistent problems of porcine IVP has been polyspermy during *in vitro* fertilization (IVF), a phenomenon in which the oocyte is penetrated by more than one spermatozoon [9]. The most effective method to reduce polyspermy reported until now is the incubation of oocytes in oviductal fluid (OF) before IVF for 30 min [10]. This incubation causes a rise in the resistance of the zona pellucida (ZP) to the sperm binding and, consequently increases by 10 times the percentages of monospermy compared with oocytes that had not been incubated in oviductal fluid [10]. A second major problem in pig IVP is the low percentage of zygotes that develop to the blastocyst stage *in vitro*. The use of swim-up as the preferred method for sperm selection, as well as the supplementation of IVF and embryo culture media with OF and uterine fluid (UF) as major protein sources, represents an important advance, allowing a final blastocyst yield of up to 40% [11]. Both Coy and Cánovas’ studies [10, 11] demonstrated that the imitation of the physiological microenvironment, achieved by placing the oocytes in a medium close to that in which they would naturally find themselves after ovulation, is an effective strategy to improve the IVP results. However, the best proof of the success of any *in vitro* embryo production system is that obtained after the transfer to recipient females, where not only the percentages of pregnancies but also the health of the offspring after birth are parameters to take into account. Until now, each of the techniques involved in assisted reproduction, as well as the culture media used and the manipulation of gametes and embryos, have been associated with lower implantation and pregnancy rates, difficulties in farrowing, fetoplacental anomalies, perinatal mortality, low birth weight (LBW), and a higher predisposition to suffer rare imprinting disorders [12–17]. However, little is known about the molecular mechanisms behind these problems.

In this sense, it has been also shown that alterations in the expression of different genes like lumican (*LUM*) or vimentin (*VIM*), which play an important role in the development of the cardiovascular system [18–20], could compromise placental functionality, but no references exist, up to our knowledge, demonstrating a direct impact of pig IVP on such alterations. On the other hand, imprinting genes, such as the paternally expressed gene 3 (*PEG3*), highly expressed in embryos and placenta, have been related with fetal growth and nurturing behaviors apart from its tumor suppressor activity [21], but, again, no data about its possible variations derived from IVP procedures are available in pigs.

For these reasons we hypothesize that the source of protein (natural or artificial) in the culture media where the embryos are produced is a factor influencing the IVP outcomes and the further ability of the embryo to develop to term. Thus, the main objective of this work was to determine the impact of the protein source of the culture media on the *in vitro* embryo development, the pregnancy and parturition outcomes, and the placental and umbilical cord molecular traits using the pig as model. To test our hypothesis, it was necessary to analyse the full-term development of embryos obtained using reproductive fluids (RF) as additives in the culture media versus those embryos obtained with the standard protocols (bovine serum albumin (BSA) as the only protein source of the culture media) and, simultaneously, contrasting the data with those obtained by AI. Doing this, and monitoring later the mid and long-term development of the offspring, it will be possible in the future to find out the phenotypical and molecular differences in the IVP-derived offspring that could represent a major risk of future pathologies in adulthood.

## Methods

Unless otherwise indicated, all chemicals and reagents were purchased from Sigma-Aldrich Chemical S.A. (Madrid, Spain). Porcine follicular fluid (FF), OF and UF were provided by EmbryoCloud (NaturARTs®, Murcia, Spain).

### Ethics

The experimental work performed in this study was submitted to evaluation by the CEEA (Comité Ético de Experimentación Animal) from University of Murcia. After approval, authorization from “Dirección General de Agricultura, Ganadería, Pesca y Acuicultura” – Región de Murcia- nr A13170706 was given to perform the experiments with animals.

### Animals

Experiments were performed in collaboration with a commercial farm (Cefu S.A., Murcia, Spain). Crossbred sows (Landrace × Large White) with the same genetic line were used as recipients (0–9 parities). All animals were housed and fed under the same conditions and water was provided ad libitum.

Weaning was used for estrous synchronization, and sows that showed signs of being clearly in heat 4–5 days after weaning were selected as recipients for embryo transfers. Estrous detection was performed by exposing a mature vasectomized boar to stimulate the estrous expression of the sow and applying the back-pressure test. Those sows that remained immobilized under such pressure were considered in heat.

The synchrony of the recipients (*n* = 23) was between − 24 and − 48 h regarding the *in vitr*o produced embryos [22]. Animals that were inseminated at the same farm, 0 and 24 h after the onset of estrous, with semen from the same boar used for IVP, were used as control group (AI group, *n* = 4).

### Oocyte collection and* in vitro* maturation

Ovaries from prepubertal crossbred gilts (Landrace × Large white) were obtained at the slaughterhouse and transported at 38.5 °C to the laboratory in saline solution containing 100 mg/mL kanamycin sulfate. They were subsequently washed at the same temperature, once in 0.04% (w/v) cetrimide solution and twice in saline solution. Cumulus cell-oocyte complexes (COCs) were collected from antral follicles between 3 and 6 mm diameter and washed twice with Dulbecco’s PBS (DPBS) supplemented with 1 mg/mL polyvinyl alcohol (PVA) and twice more in maturation medium previously equilibrated for a minimum of 3 h at 38.5 °C under 5% CO_2_ in air. Maturation medium was NCSU-37 [23] supplemented as previously described [11].

COCs with complete, compact and dense cumulus oophorus and uniform ooplasm [24] were selected, and groups of 50–55 COCs were cultured in 500 μL maturation medium for 22 h at 38.5 °C under 5% CO_2_ in air. After culture, oocytes were washed twice in fresh maturation medium without dibutyryl cAMP, eCG and hCG and cultured for an additional time period of 20–22 h [25].

### *In vitro* fertilization

After 44 h of maturation, cumulus cells were partially removed mechanically by pipetting and mature oocytes were washed twice in TALP medium [11] previously equilibrated at 38.5 °C under 5% CO_2_.

Mature oocytes were divided in two groups according to the IVF medium supplementation: Bovine serum albumin (C-IVP) and Reproductive fluids (RF-IVP). The medium for C-IVP was supplemented with 3 mg/mL fatty acid-free bovine serum albumin (BSA-FAF), while 3 mg/mL BSA-FAF and 1% (v/v) POF from the late follicular phase of the estrous cycle (NaturARTs® POF-LF, EmbryoCloud, Murcia, Spain) were added to the RF-IVP medium. Groups of 50 oocytes per well were placed in 4-wells multidishes containing 250 μL TALP medium.

Ejaculated spermatozoa from a fertility-tested boar (1 year old, same animal used for AI) were transported to the laboratory and diluted 1:5 in Beltsville thawing solution (BTS [26],) remaining stored at 16 °C for 24 h until IVF. At 24 h, the sperm solution was centrifuged (300 ×*g*, 10 min) and the supernatant was collected. For sperm selection, 1 mL of semen was laid at the bottom of a conical tube, below 1 mL tempered NaturARTs® PIG sperm swim up medium (EmbryoCloud, Murcia, Spain) supplemented with 0.5% BSA or 1% (v/v) POF from late follicular phase of the estrous cycle (NaturARTs® POF-LF, EmbryoCloud, Murcia, Spain) for C-IVP and RF-IVP groups, respectively. The swim up tubes were incubated for 20 min at 37 °C placing the tube at a 45° angle. After that incubation, 0.5 mL from the top of the tube were recovered with and automatic pipette and spermatozoa concentration and motility were assessed by an experienced technician. Afterwards, and according to sperm assessment, sperm were diluted in 250 μL TALP medium in a concentration (5–15) × 10^3^ spermatozoa/mL. Spermatozoa and oocytes were cocultured for 20 h at 38.5 °C and 5% CO_2_. Putative zygotes were pipetted to remove supernumerary sperm attached to the zona pellucida, transferred to embryo culture medium and a small sample of each group was taken to assess the fertilization rates by fixing and staining the putative zygotes as previously described [10].

### Embryo culture

The embryo culture medium was NCSU-23 [23] supplemented as previously described [11].

Twenty hpi, putative zygotes were washed once in NCSU23-A and transferred to culture dishes containing the same medium with or without 1% (v/v) POF from the early luteal phase (NaturARTs® POF-EL, EmbryoCloud, Murcia, Spain) for the RF-IVP and C-IVP, respectively. Wells with embryos were covered with parafin oil (Nidoil, Nidacon, Sweden). Putative zygotes were incubated at 38.5 °C under 5% CO_2_ and 7% O_2_.

After 48 hpi, the cleavage was assessed under the stereomicroscope and the 2–4 cell stage embryos were washed and transferred to NCSU23-B supplemented or not with 1% (v/v) of PUF from mid luteal phase of the estrous cycle (NaturARTs® PUF-ML, EmbryoCloud, Murcia, Spain) for the RF-IVP and C-IVP, respectively. Each well was covered with Nidoil and embryos remained in culture (38.5 °C, 5% CO_2_ and 7% O_2_) until day 5 or 6 (120–144 hpi).

### Surgical embryo transfer by paralumbar laparo-endoscopy single-site

**Fig. 1:**
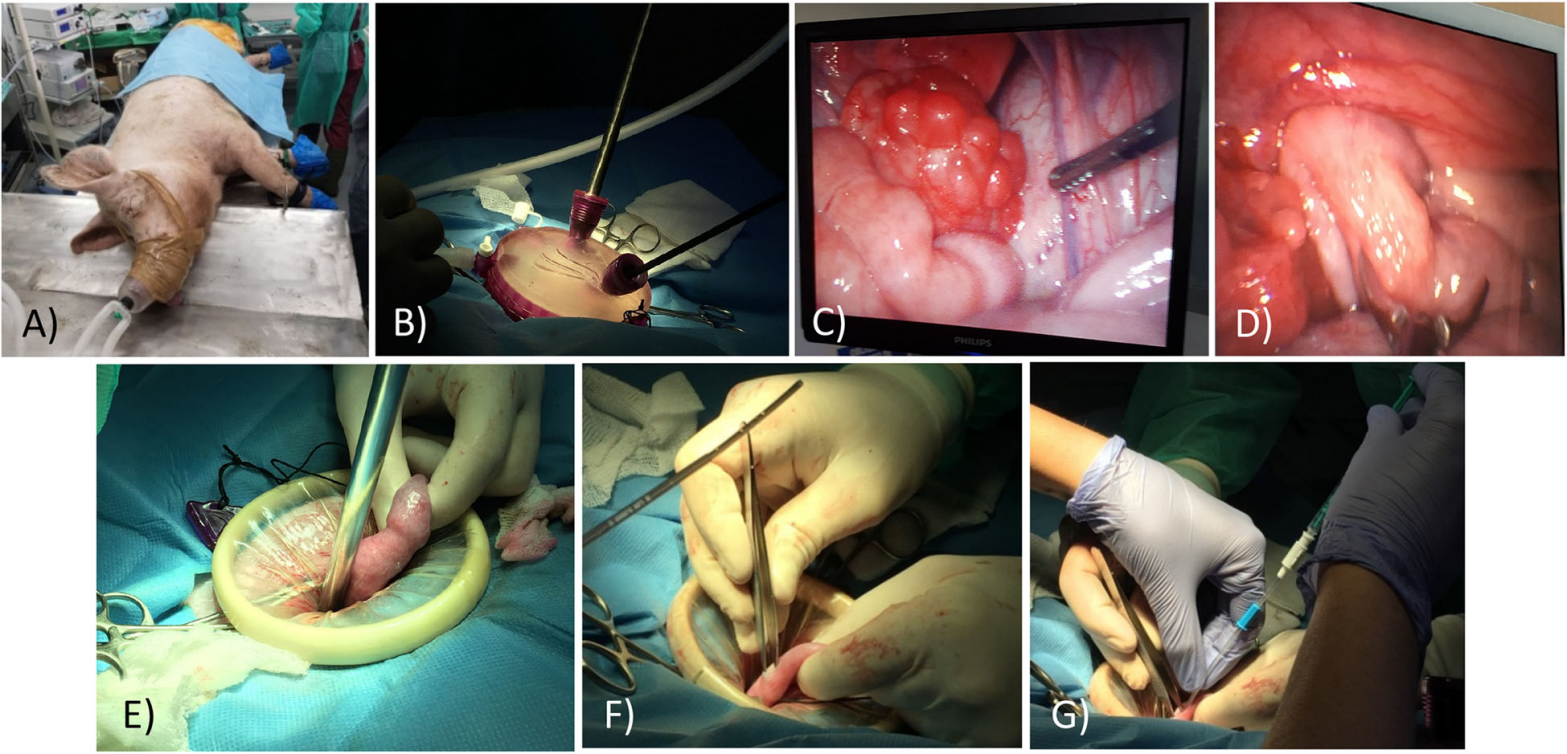
Surgical embryo transfer. Paralumbar laparo-endoscopy single-site (LESS) assisted approach with a monoport device. **a** Anaesthetized sow prepared for laparoscopy. **b** After placement of the monoport cap pneumoperitoneum was used to set up two working ports, one for camera and another for forceps. **c** Laparoscopic view of the ovary. **d** Laparoscopic view of how the uterine horn is grasped with atraumatic forceps. **e** Externalization of the uterine horn after removal of the monoport cap. f Uterine horn wall puncture with forceps. g Intrauterine deposition of embryos

Considering day 0 as the onset of estrous, on days 4–5, recipients were anesthetized, after a 24-h fast. Sedation was done by administration of ketamine (10 mg/kg IM, Imalgene® 1000, Boehringer Ingelheim Animal Health, Merial, France), medetomidine hydrochloride (0.02 mg/kg IM, Domitor®, Orion Pharma, Finland) and midazolam (0.2 mg/kg IM, Dormicum®, Roche, Switzerland). Then, anesthetic maintenance and analgesia were reached through the administration of isoflurane (2–3% O_2_ Isoflurin, Fatro Iberica, Spain) and buprenorphine (0.01 mg/kg IM, Bupredine®, Fatro Iberica, Spain), respectively (Fig. 1a).

To perform the surgical approach, the incision area was shaved, washed and disinfected previously with chlorhexidine (1% Desinclor, Imark, Madrid, Spain). The technique used to perform the embryo transfer was a paralumbar laparo-endoscopy single-site (LESS) assisted approach with a monoport device (GelPOINT® Advanced Access Platform, Applied Medical, Rancho Santa Margarita, California, USA) formed by a cap of gel and two flexible rings connected by a plastic membrane (Fig. 1b). Sows were placed in lateral recumbence and a mini-laparatomy of 3–4 cm, approximately, was performed. To stabilize the incision in the abdominal wall, one of the flexible rings was placed inside, remaining the second adhered to the abdominal wall externally. Monoport cap was placed on it and two trocars were used to insert the endoscope and a non-traumatic laparoscopy forceps through them into abdominal cavity which was then filled with CO_2_ pneumoperitoneum of 8–10 mmHg (Fig. 1b) to facilitate the visualization of the reproductive organs and the handling of these to locate the ovaries. Then, they were assessed to confirm ovulation and presence of corpora lutea (Fig. 1c). Later, one of the uterine horns was grasped with the forceps and taken to the port opening (Fig. 1d, e). Next, pneumoperitoneum and monoport cap were removed to allow a gentle manipulation and the end of the uterine horn was punctured with toothless micro Adson forceps allowing, through a small opening, the introduction of the catheter and deposition of embryos into the lumen of uterine horn (Fig. 1f, g).

On days 5 or 6 after IVF, embryo morphology was evaluated under stereomicroscope before being transferred to recipients. Then, a commercial medium (BO-Transfer, IVF Bioscience, Denmark) was used to wash and transport the embryos from the laboratory to the farm in a portable incubator at 38.5 °C. Time between embryo transport and transfer did not exceed 45–60 min.

Embryos were loaded into an embryo-tested, 1 mL syringe (Non-toxic syringe, COOK® Medical, Ireland) using the following sequence of aspiration: 0.1 mL of BO-Transfer medium (IVF Bioscience, Denmark), 0.1 mL air, 0.1 mL of BO-Transfer medium with embryos, 0.1 mL air, and finally 0.1 mL BO-Transfer medium. Then, the syringe was attached to a catheter (Emtrac Delphin catheter, Gynetics® Lommel, Belgium) and the embryos were introduced within the uterine horn (explained in detail above). An additional 0.3 mL of BO-Transfer medium was used to wash the catheter. Finally, the catheter was removed and re-washed with the same medium on a culture dish to verify that no embryos remained inside.

The number of embryos per transfer and sow ranged between 26 and 93. Finally, the surgical incision was sutured in three planes and a dose of amoxicillin (20 mg/kg IM Clamoxyl LA®; Pfizer, Madrid, Spain) was administrated.

### Pregnancy diagnosis and collection of farrowing-related data

Pregnancy was diagnosed by ultrasonography 21–26 days after the onset of estrus. All sows were housed in gestation crates located in a parturition unit at the farm and few days before giving birth, sows were monitored continuously by a camcorder to observe possible signs of parturition. At the day of farrowing, gestation length, farrowing rate, survival rate and litter size were registered.

Piglets were weighed using a digital hanging scale and, immediately after, they were placed with their mother.

The placentas were individually identified as previously described [27]. In brief, a double ligature was made to each umbilical cord as the piglets were expelled, sewing a numbered tag to the end of the cord that would be retracted into the vagina. After delivery, an umbilical cord sample was taken out of each piglet and the remaining was disinfected with chlorhexidine (2% Clorhexidine, Lainco, Barcelona, Spain). .

Three different placental parameters were analysed: weight (g), area (cm^2^) and efficiency (g/g). For the first, each of the previously identified placentas was weighed using a digital hanging scale. Placentas were displayed on paper to trace their contour and their surface area was analysed by ImageJ 1.52a software (National Institute of Health, USA), subsequently multiplying the area by two. Finally, the placental efficiency was calculated as the ratio of birth weight to placental weight and reflects grams of fetus produced per gram of placenta.

### RNA isolation and quantitative real-time PCR

Genes with different functions related to imprinting, angiogenesis, transcription, and glucose transport [28] were selected to study their differential expression, as shown in supplementary Table 1.

Total RNA was extracted from two different tissues (placenta and umbilical cord) of the two males and females with the highest and lowest weight from each litter, using Trizol® (Invitrogen, United Kingdom).

Collected tissues were washed with PBS, and immediately immersed and stored in liquid nitrogen until further use. To process the tissues, samples were defrosted at room temperature and afterwards immersed in Trizol reagent. Proteins were removed with chloroform extraction, and the RNA pellets were washed once with isopropyl alcohol, followed by a wash with 70% ethanol solution prepared with RNAse, DNAse-free water (Gibco, Invitrogen, United Kingdom). The total RNA pellets were reconstituted in RNAse free water (Gibco, Invitrogen, United Kingdom). A microspectrophotometer (NanoDrop 2000c, Thermo Scientific, USA) was used to quantify the extracted RNA (ng/μL).

The single-strand cDNA synthesis by RT-PCR was performed using SuperScript™ III Reverse Transcriptase kit (Invitrogen, United Kingdom) according to manufacturer’s protocol. Briefly, a reaction mixture consisting of 1× iScript Reaction Mix, 1 μL iScript Reverse Transcriptase, RNA template (1 μg total RNA) and nuclease-free water was prepared, for the final volume of 20 μL. The reaction was performed under the following conditions: 25 °C for 5 min, 42 °C for 30 min, and 85 °C for 5 min.

The expression of selected genes (supplementary Table 1) was measured by quantitative real-time PCR (q-PCR) on a CFX96 Touch System (Bio-Rad Laboratories, USA). SYBR® Green PCR Master Mix (Applied Biosystems™, USA) was used to perform real-time PCR, according to the manufacturer’s indications.

Reactions were prepared for the final reaction volume of 25 mL, using a dilution of 1/20 of cDNA products, 1 μmol/L specific primer (primer sets listed in Supplementary Table 1), 1× iQ SYBR Green Supermix and nuclease-free water. The primers for each selected gene were designed using Primer3 web application (http://primer3.ut.ee/).

The cycling conditions followed were an initial denaturation at 95 °C for 10 min followed by 40 cycles of denaturation at 95 °C for 10 s, annealing at 60 °C for 10 s with an extension at 72 °C for 12 s, and finally a melting curve consisting of 95 °C for 20 s and 65 °C for 20 s. The gene expression study for each gene per sample was performed in triplicate, and reactions were performed on the C1000 Touch Thermal Cycler (Bio-Rad Laboratories, USA). The fold change in gene expression of selected target genes relative to the housekeeping gene β-actin gene (ActB) was calculated by the Livak (2 ^-∆∆CT^) method [28].

### Statistical analysis

Normality tests (Shapiro-Wilk, *P* > 0.05) were performed for each parameter and normal data were analysed by parametric test *t*-test or one-way ANOVA followed by Tukey’s multiple comparison test. Data that did not follow normality were analysed by non-parametric Mann Whitney U or Kruskal-Wallis test, followed by Dunn’s multiple comparison test. Values of *P* < 0.05 were considered significantly different. The results are presented as mean ± SD (standard deviation), unless otherwise specified. Software used was GraphPad Prism, version 8.4.2 for Windows (GraphPad Software, San Diego, California USA).

### Experimental design

**Fig. 2:**
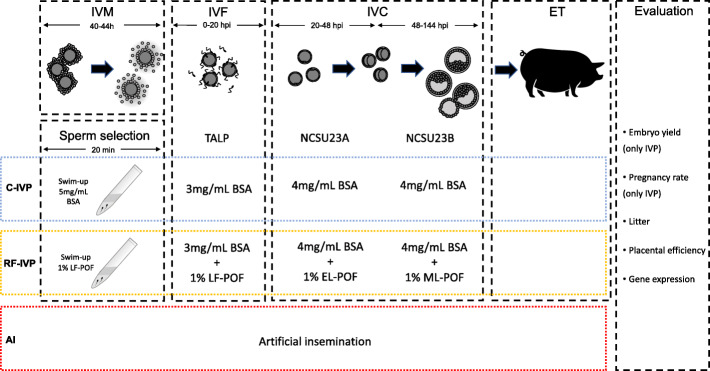
Schematic representation of the experimental design in the study

Embryos were produced by *in vitro *fertilization, cultured in media with different protein sources and surgically transferred to recipients at the blastocyst stage (Fig. 2). Outcomes of the embryo development *in vitro* and pregnancy rates were analysed. A control group of piglets born by AI in the same farm from sows of the same genetic background was used. Parturition issues, birth weight, placental parameters (placental weight, surface area and placental efficiency) as well as placental and umbilical gene expression were evaluated in the three groups (Fig. 2).

## Results

### *In vitro* produced embryos

Table 1 shows the cleavage rate and blastocyst yield obtained after the *in vitro* embryo production by using or not reproductive fluids as additives for the culture media. No significant differences were found (*P* < 0.05) between both groups for these parameters.

**Table 1:** Cleavage rate, blastocyst yield and total number of embryos transferred after IVP with (RF-IVP) or without (C-IVP) reproductive fluids as an additional source of protein to the culture media. Data are expressed as mean ± SD

Group	Blastocyst, *n*	Cleavage, %	Blastocyst/Cleavage, %	Blastocyst/Oocyte, %
RF-IVP	724	69.41 ± 6.97	21.00 ± 3.93	14.66 ± 3.76
C-IVP	660	66.92 ± 18.36	22.55 ± 8.20	13.99 ± 2.90

### Pregnancies and farrowing rates

Five of 13 recipients (38.5%) and four of 11 recipients (36.4%) became pregnant in the RF-IVP and C-IVP groups, respectively (supplementary Table 2). The remaining sows returned to estrus at 19–22 days after being transferred.

The farrowing rate was 80% in the RF-IVP group, since abortion in one of the sows occurred after being positively diagnosed at first. On the other hand, all the pregnancies in the C-IVP group came to term, with the farrowing rate being 100%. No significant differences were found between groups although induction of parturition was necessary in 1 animal from the AI group as well as in 2 and 4 animals from the C-IVP and RF-IVP groups, respectively.

As for the synchrony between the developmental stage of the embryos and the day of reproductive cycle of mothers, two of the pregnant recipients from RF-IVP group and one from C-IVP group presented a synchrony of − 24 h regarding the embryos; conversely, 3 of the pregnant recipients in the RF-IVP group and 3 of those in C-IVP group were at − 48 h regarding the embryos (Supplementary Table 2).

All the resulting positive pregnancies corresponded to the transfer of blastocyst and/or hatched blastocysts embryo stages (Supplementary Table 2).

Gestation period did not vary significantly between groups, although a tendency to shorter gestational lengths was observed in RF-IVP vs. AI group (*P =* 0.0539).

In total, 105 live piglets (59 females and 46 males) from 12 litters (4 per group) were born, being 58 of them from the AI group, 29 from C-IVP and 18 from RF-IVP (Table 2). The average number of live piglets born per litter was higher in the AI group than in both RF-IVP and C-IVP groups (14.50 ± 6.40, 4.75 ± 1.71; and 7.5 ± 2.52, *P* < 0.05, for groups AI, C-IVP and RF-IVP, respectively). None of them presented morpho-anomalies. One out of 19 animals from RF-IVP group, as well as 1 out of 30 from C-IVP were born dead, while no deaths were counted for the AI group at birth.

**Table 2:** Total piglets, litter size and piglets’ sex derived from RF-IVP, C-IVP or AI

Groups	RF-IVP	C-IVP	AI
Total No. of piglets born (live-born piglets)	19 (18)	30 (29)	58 (58)
Males (live-born males)	11 (10)	11 (11)	25 (25)
Females (live-born females)	8 (8)	19 (18)	33 (33)
Litter size			
Total litter size (males + females)	4.75 ± 1.71^a^	7.50 ± 2.52^ab^	14.50 ± 6.40^b^
Males	2.75 ± 0.50	2.75 ± 1.71	6.25 ± 3.69
Females	2.00 ± 1.41^a^	4.75 ± 3.77^ab^	8.25 ± 4.03^b^

The number of live born piglets appears in brackets

^a-b^Values in the same row with different superscripts are significantly different (*P* < 0.05)

### Birth weight and placental parameters

Table 3 displays birth weight, placental weight, surface area and efficiency for all our three groups. When birth weight was analysed, significant differences (*P* < 0.05) were found between piglets born from AI group and those derived from IVP (RFIVP, C-IVP). Similarly, placentas were heavier for both RF-IVP and C-IVP when compared to AI placentas (*P* < 0.05) but the surface area was only significantly higher in C-IVP than AI placentas (*P* < 0.05). Additionally, RF-IVP placentas showed significantly lower efficiency when compared with the AI group (*P* < 0.05).

**Table 3:** Birth weight, placental weight, surface area and placental efficiency in piglets derived from *in vitro* produced embryos with (RF-IVP) or without (C-IVP) reproductive fluids and from artificial insemination (AI)

Groups	RF-IVP	C-IVP	AI
Birth weight, g, (*n*)	1381.0 ± 425.4^a^ (*n* = 19)	1524.0 ± 340.1^a^ (*n* = 29)	1170.0 ± 281.7^b^ (*n* = 58)
Total No. of placentas, *n*	*n* = 17	*n* = 13	*n* = 23
Placental weight, g	189.40 ± 74.20^a^	182.30 ± 46.93^a^	127.40 ± 55.29^b^
Placental surface area, cm^2^	1700.00 ± 473.00^ab^	2110.00 ± 763.40^b^	1561.00 ± 348.50^a^
Placental efficiency, g/g	7.42 ± 1.67^b^	8.99 ± 2.62^ab^	10.92 ± 3.54^a^

^a-b^Values in the same row with different superscripts are significantly different (*P* < 0.05)

### Gene expression levels in placenta and umbilical cord

**Fig. 3:**
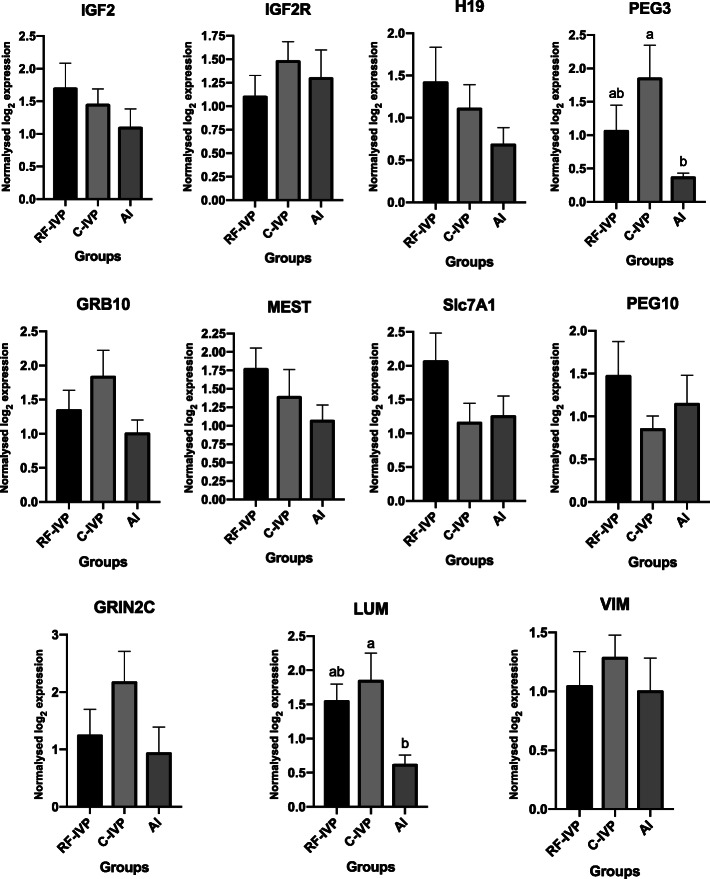
Expression of selected genes from placental tissue derived from the three groups of piglets analysed: RF-IVP (*n* = 9), C-IVP (*n* = 9) and AI (*n* = 10). All data are presented as the mean ± SEM. Different letters (a, b, c) indicate significant differences (*P* < 0.05)

When expression of specific genes was assessed in placenta, *PEG3* showed a significantly increased expression level in C-IVP compared with AI (*P* < 0.05) (Fig. 3). Similarly, *LUM* was more expressed in C-IVP compared with AI (*P* < 0.05) (Fig. 3). However, no differences were found in the transcription levels for the rest of genes analysed (*P* > 0.05) (Fig. 3).

**Fig. 4:**
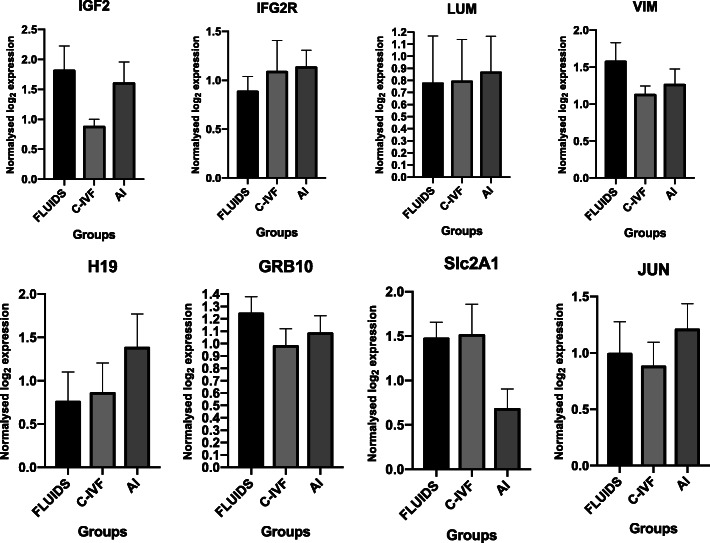
Expression of selected genes from umbilical cord tissue derived from the three groups of piglets analyzed: RF-IVP (*n* = 8), C-IVP (*n* = 9) and AI (*n* = 9). All data are presented as the mean ± SEM

No differences were found regarding umbilical cord expression levels (*P* > 0.05) (Fig. 4).

## Discussion

In animal species, where infertility factors of selected breeders can be discarded, it is well documented that changes induced by exposure of embryos to suboptimal conditions can derive in phenotypic defects [17, 29, 30]. Indeed, in cattle and sheep it has been noted a higher incidence of embryo loss, higher pregnancy length, large body size and birth weight in ART-derived gestations [31]. In addition, the large offspring syndrome described in cattle is an imprinting disorder with similar features to Beckwith-Wiedemann Syndrome in humans, such as macrosomia, macroglossia or visceromegaly [32, 33]. Other anomalies observed in IVP-derived cattle due to an overgrowth in late gestation are a higher incidence of dystocia, hydrallantois, and neonatal mortality [29, 30].

Even though numerous studies in cattle have enabled insights into the impact derived from ART, the information is almost inexistent in porcine species. Thus, it becomes relevant to describe the phenotypical consequences in the offspring and, if possible, to understand the mechanisms involved in potential anomalies derived from ART-derived piglets.

Results from the present study showed that, when the reproductive fluids were introduced in the culture media at the different steps of the *in vitro *production system, the percentages of cleavage and blastocysts were similar to those obtained in absence of fluids. As the use of reproductive fluids is not yet a common practice in the field, the only work previously published to compare with is that from our own group [11]. In that study, the results showed a 5% higher cleavage rate in the control group than in the group using reproductive fluids, although both values were below the values in the present study (lower than 50% in Cánovas et al. [11] for both groups vs. higher than 65% in the present study for both groups). As in the present study, the percentage of blastocysts in Cánovas et al. study [11] was not different regarding the presence or absence of reproductive fluids. These results confirm what was previously proposed about the low impact that using reproductive fluids has on the final number of embryos obtained. However, at this point, the higher quality of the embryos described by Canóvas et al. [11] cannot be confirmed because no other parameters were analysed in the present study. Instead, we transferred most of the produced embryos to investigate their ability to implant and develop to term.

First data sets that called to our attention regarding the pregnancy rate after transfers was the higher percentage (> 35% in both, C-IVP and RF-IVP groups) of positive pregnancies with the* in vitro *produced embryos compared to those in our previous study using* in vivo* produced embryos (27%, Paris-Oller et al., unpublished). Such apparent paradox can be explained because the recommended range of asynchrony between donor and recipients for pig ET must be between 0 and (−) 48 h [22, 34] and this was the rule we followed in the present study, but not in our previous one. Thus, we assume this was the reason for the higher pregnancy and parturition outcomes obtained here. Also the use of a minimally invasive procedure based on a LESS approach might have influenced positively the pregnancy rate because, as demonstrated in previous works [35, 36], the trauma to the uterus was minimal and the full recovery of the animal after procedure significantly reduced compared to laparotomy. Despite this, the percentage of non-pregnant animals after embryo transfer in our study was still higher than 64% in both groups. As it has been recently proposed, this could be associated with a dysregulation of pro- and anti-inflammatory cytokine levels in recipient sows that, in turn, induce embryonic mortality [37]. However, many other factors related to either the quality of the embryos or the recipient’s status and age could have been affecting these rates of unsuccessful transfers [38]. On the one hand, and as for the own ET procedure, the reported levels of embryonic mortality by using non-surgical procedures are approximately of 70% [39, 40], while using surgical laparoscopic procedures, Wieczorek et al. [41] reported 50% of successful pregnancies but those were after transferring *in vivo* produced embryos. This is, on the other hand, other crucial factor to be considered because all the above referred rates derive from embryos produced *in vivo *and ours were produced *in vitro*. In fact, for most of the researchers, the transfer of embryos produced *in vivo* (but not *in vitro*) is the only one with “possible short-term use in pig production” [38] although, from our results, this statement should probably be reconsidered since our pregnancy rates are comparable to those obtained transferring* in vivo* collected embryos.

Actually, the high farrowing rates in our experiment, with only one miscarriage happening in the RF-IVP group at day 24 post-transfer (which is regarded as the time frame for implantation), can be considered good indexes of the quality of the embryos transferred, although more studies with higher sample sizes are needed to confirm this statement.

As for the gestation period, it is an index depending on the litter size, but it is well known that some other factors such as farm, parity, number of inseminations or genetic line can affect it [42]. While short gestation lengths are associated with higher stillborn, long pregnancies are not desired by the farmers and the advantages of inducing and attending farrowing compared to letting the sows go on their own are a matter of current debate [42]. In our experience, delivery inductions with oxytocin were necessary in 1, 2 and 4 animals from AI, C-IVP and RF-IVP groups respectively due to delays between the birth of the first and following piglets, but not because the parturition did not start spontaneously. Our presence during delivery, in order to take the individualized umbilical cord and placenta samples, could have acted as an additional stressor contributing to the delays and, consequently, we cannot affirm at this point if such problems were related to the embryo transfer procedure, the embryo source, or our own presence.

The litter size, in our case, was not a factor that affected gestation length in IVP pregnancies because the sow with the longest period (121 days) delivered only 5 piglets while the sow with the highest litter size (10 piglets) delivered at day 115. Similarly, the fact that embryos were *in vitro* produced was not a factor affecting gestation length because the AI animals showed similar periods of pregnancy length. 

Studies in cloned piglets showed several abnormalities in both placenta and umbilical cord [27], but data regarding IVF piglets are limited. In the present study, expression level of key genes related with imprinting, angiogenesis or glucose transport were analysed in placenta and umbilical cord. Fetal weight was found to be proportional to placental weight in several studies [43–45] whereas reduced placental weight has been reported in somatic cell nuclear transfer derived piglets vs those produced by AI [27]. However, while this parameter has been studied in pig with productive purpose [46, 47], in other species such as mice, changes in placental weight have been related to exposure to stressors during *in vitro* production [48, 49]. Our results show significant differences in the placental weight between the experimental groups (C-IVP and RF-IVP) and AI. Nonetheless, placental area is considered a better marker for postpartum piglets’ viability. Placental area is also highly associated with birth weight [50] and lower placental area was found in piglets dead at weaning vs piglets alive at weaning [47]. In our study, both RF-IVP and C-IVP group displayed larger placental area than AI placentas. However, it should be noted that both IVP groups had higher birth weights (data not shown) while the litter size was smaller than AI. This is in accordance with the negative association reported between birth weight and placental weight with live litter size [47].

Complementary to placental area, placental efficiency (PE) could provide information about placental functionality, with high PE values associated with greater nutrient transport capacity. PE was significantly decreased in the RF-IVP vs. AI. Nonetheless, this parameter shows natural variation in pigs and between breeds. Even within the same litter, PE can vary significantly, with piglets having similar birthweight but very different placental weight (up to 25%) [46, 51]. Moreover, the use of PE as a selection tool to increase litter size is debatable, because an increase in litter size could result in reduced birth weight and higher mortalities. It is controversial regarding welfare animal conditions, even in the hypothetic situation that global outcome would remain beneficial.

As for the molecular analyses, it is known that placental nutrient transport capacity is related with gene expression of transporter genes. In mice, *Slc2a1* *(*glucose transporter) and *Slc38a2 (*amino acid transporter) were upregulated in the lightest placentas, confirming that placentas with high PE adapt and increase nutrient transport efficiency. Contrary, *SLC7A1, *a cationic amino acid transporter, was found negatively related to PE [51]. Placentas in the RF-IVP group, with the lowest PE, showed double *SCL7A1* expression than C-IVP or AI, but these differences were not statistically significant, perhaps by the reduced number of samples. In the umbilical cord, *SCL2A1* expression did not show significant differences, but there was a tendency (*P* = 0.0502) with RF-IVP and C-IVF showing expression values over AI.

*LUM* and *VIM* genes, whose function is related to the angiogenesis process, were selected as possible markers for placental functionality because it was previously described that children born by IVF showed higher levels of *LUM* and lower levels of *VIM* expression in their umbilical vein endothelia cells than children naturally conceived [18]. Altered expression of these proteins would explain, according with [18], the cardiovascular dysfunction and vascular remodelling occurring in IVF offspring. Our data, however, did not show differences in *VIM* or *LUM* expression between AI, RF-IVP and C-IVP in umbilical cord samples, suggesting that our IVP system could not be affecting in such way the cardiovascular function of the offspring. However, placentas from AI piglets showed significantly lower *LUM* expression than those from C-IVP piglets, whereas those from RF-IVP were not different from AI. This result could be suggesting a protective effect of reproductive fluids on the altered expression of *LUM*, whose consequences in the short-term development of the offspring should be further studied.

As for *PEG3*, it is well known that placenta from ART-derived animals exhibits higher probability of perturbations in genomic imprinting (mouse [52], pig [27], bovine [32], human [53]). Under our experimental conditions, *PEG3* expression was upregulated in C-IVP embryos vs. AI in placenta but the same did not happen for RF-IVP group, that was similar to AI. Even though P*EG3* expression level is sexually biased, with two-fold higher levels in males than females [54], this cannot be the explanation for our results because the proportions of males was 39% in C-IVP piglets. Again, this finding could be of high interest and could confirm the beneficial effect of reproductive fluids on the phenotype of the offspring, but additional studies with higher sample sizes are still necessary to confirm this hypothesis.

Finally, it is worth mentioning that all the animals derived from the present study were kept alive and studies of their growth, glucose metabolism, and haematological and biochemical profiles along their lives are being currently undergone.

## Conclusions

From results presented and discussed above, it has been shown that the use of reproductive fluids to produce *in vitro-*derived pig offspring gave similar outcomes to those derived from a standard protocol, in terms of embryo yield, pregnancy rates and farrowing rates. However, litter size in the ART groups was still below the average litter size after artificial insemination. Even though placental weight displayed higher values in both IVP groups than AI placentas, placental surface was higher for C-IVP than for AI piglets, and placental efficiency was lower for RF-IVP vs. AI offspring. Gene expression at placental tissue showed differences for *PEG3* and *LUM* in C-IVP group compared to AI group, but not in RF-IVP group. Whether or not these differences may influence the short and long-term life of the piglets, is a question under research in these animal colony at present.

## Supplementary Information

**Additional file 1: Table S1.** Primers sequences for quantitative real-time PCR.

**Additional file 2: Table S2*.*** Pregnancy and farrowing results after transfer of *in vitro* produced embryos produced with/without reproductive fluids.

## Abbreviations

ART: Assisted reproductive technologies
AI: Artificial insemination
ET: Embryo transfer
IVP: *In vitro* production
IETS: International Embryo Technology Society
IVF: *In vitro* fertilization
OF: Oviductal fluid
UF: Uterine fluid
LBW: Low birth weight
RF: Reproductive fluids
BSA: Bovine serum albumin
COCs: Cumulus cell-oocyte complexes
PVA: Polyvinyl alcohol
DPBS: Dulbecco’s PBS
IM: Intramuscular administration
hpi: Hours post insemination (*in vitro*)
POF: Porcine oviductal fluid
BTS: Beltsville thawing solution
PE: Placental efficiency
