# Supplementary material for: Reproductive fluids, used for the in vitro production of pig embryos, result in healthy offspring and avoid aberrant placental expression of PEG3 and LUM
Source: J Anim Sci Biotechnol. 2021 Feb 15;12:32. doi: 10.1186/s40104-020-00544-0 (PMC7883450; doi:10.1186/s40104-020-00544-0)
Supplement: Supplementary file 1 — Additional file 1: Table S1. Primers sequences for quantitative real-time PCR. [file 40104_2020_544_MOESM1_ESM.docx]

**Supplementary Table 1.** Primers sequences for quantitative real-time PCR

| Gene | Functional role | Description | Primer sequence (5´→3´) | Product size, bp | GenBank ID |
| --- | --- | --- | --- | --- | --- |
| *IGF2* | Imprinted gene | Insulin like growth factor 2 | Forward: ACACCCTCCAGTTTGTCTGC  Reverse: AAGCAGCACTCTTCCACGAT | 100 | X56094.1 |
| *IGF2R* | Imprinted gene | Insulin like growth factor 2 receptor | Forward: GCCGGCGAATACACCTATTA Reverse: CATCTTCAACACCCCGTTCT | 180 | [NM_001244473](http://www.ncbi.nlm.nih.gov/nuccore/NM_001244473) |
| *H19* | Imprinted gene | Imprinted maternally expressed | Forward:GGCCGGAGAATGGGAAAGAAGG  Reverse: CGCAGTGCTGCGTGGGAACG | 148 | AY044827.1 |
| *PEG3* | Imprinted gene | Paternally expressed gene 3 | Forward: GTCGCAGAAGAGTCACACCA  Reverse: AGCTGCGAAGAACAGACCTC | 128 | XM_021095134.1 |
| *GRB10* | Imprinted gene | Growth factor receptor bound protein 10 | Forward: CTTCCCCGAACAGATGGTTA  Reverse: CCCTTCGTGGAGCAGTAGAG | 192 | NM_001134965.1 |
| *MEST* | Imprinted gene | Mesoderm specific transcript | Forward: AAGGGACTGCGCATCTTCTA  Reverse: AGGGTCAGACCTTCCCAGAT | 125 | NM_001128471.1 |
| *SLC7A1* | Imprinted gene | Solute carrier family 7 member 1 | Forward: CATCAAAAACTGGCAGCTCA  Reverse: TGGTAGCGATGCAGTCAAAG | 194 | [NM_001012613](http://www.ncbi.nlm.nih.gov/nuccore/NM_001012613) |
| *GRIN2C* | Imprinted gene | Glutamate ionotropic receptor NMDA type subunit 2C | Forward: TGTCTGGCCTCAGTGACAAG  Reverse: GTGCATGTCACGGTAGTTGC | 122 | XM_021066670.1 |
| *LUM* | Angiogenesis | Lumican precursor | Forward: TCTGCTGGAGCTGGATCTCT  Reverse: CGCAAATGTTTGATCTTGGA | 165 | NM_001243339.1 |
| *VIM* | Angiogenesis | Vimentin | Forward: ATGCTTCTTTGGCACGTCTT  Reverse: GATTTGGACGTGCTGTTCCT | 134 | XM_005668106 |
| *PEG10* | Imprinted gene | Paternally expressed 10 | Forward: AGCGATCCCACTACCTGATG  Reverse: CGTTCCAATCCAGATCCTGT | 138 | NM_001109944.2 |
| *JUN* | Transcription | Transcription factor AP-1 | Forward: CCCAAGATCCTGAAGCAGAG  Reverse: GATGTGCCCGTTACTGGACT | 174 | NM_213880.1 |
| *SLC2A1* | Glucose transport | Solute carrier family 2 member 1 | Forward:GCAGGAGATGAAGGAGGAGAGC  Reverse: ACGAACAGCGACACGACAGT | 258 | XM_021096908.1 |
