# Supplementary material for: Reproductive fluids, used for the in vitro production of pig embryos, result in healthy offspring and avoid aberrant placental expression of PEG3 and LUM
Source: J Anim Sci Biotechnol. 2021 Feb 15;12:32. doi: 10.1186/s40104-020-00544-0 (PMC7883450; doi:10.1186/s40104-020-00544-0)
Supplement: Supplementary file 2 — Additional file 2: Table S2. Pregnancy and farrowing results after transfer of in vitro produced embryos produced with/without reproductive fluids. [file 40104_2020_544_MOESM2_ESM.docx]

**Supplementary Table 2*.*** Pregnancy and farrowing results after transfer of *in vitro* produced embryos produced with/without reproductive fluids.

| Recipient ID | Group | No. of parities | Estrous cycle day, d | Age of embryos, d | Synchrony recipient-embryos, h | Embryos transferred, *n* | Embryonic stage | Pregnancy (+/-) | Gestation period, d | Total piglets, *n* |
| --- | --- | --- | --- | --- | --- | --- | --- | --- | --- | --- |
| 1 | C-IVP | 0 | 4 | 6 | -48 | 93 | B | - |  |  |
| 2 | C-IVP | 1 | 5 | 6 | -24 | 61 | B | - |  |  |
| 3 | C-IVP | 4 | 5 | 6 | -24 | 31 | B-ExB | - |  |  |
| 7 | C-IVP | 1 | 4 | 6 | -48 | 40 | B | + | 115 | 10 |
| 5 | C-IVP | 5 | 4 | 6 | -48 | 33 | B | - |  |  |
| 6 | C-IVP | 0 | 5 | 6 | -24 | 38 | ExB | - |  |  |
| 13 | C-IVP | 3 | 4 | 5 | -24 | 34 | M-B | - |  |  |
| 15 | C-IVP | 9 | 5 | 6 | -24 | 47 | B-ExB | + | 116 | 6 |
| 17 | C-IVP | 5 | 5 | 6 | -24 | 47 | B | - |  |  |
| 19 | C-IVP | 5 | 4 | 6 | -48 | 42 | B | + | 121 | 5 |
| 22 | C-IVP | 6 | 4 | 6 | -48 | 47 | ExB | + | 115 | 7 |
| 3 | RF-IVP | 2 | 5 | 6 | -24 | 45 | M-ExB | - |  |  |
| 4 | RF-IVP | 5 | 4 | 6 | -48 | 40 | B | - |  |  |
| 5 | RF-IVP | 5 | 5 | 6 | -24 | 32 | ExB | + | 113 | 5 |
| 8 | RF-IVP | 6 | 4 | 6 | -48 | 52 | B | + | 111 | 3 |
| 10 | RF-IVP | 0 | 5 | 6 | -24 | 42 | B-ExB | - |  |  |
| 12 | RF-IVP | 5 | 5 | 6 | -24 | 41 | B-ExB | - |  |  |
| 14 | RF-IVP | 6 | 4 | 5 | -24 | 31 | B | - |  |  |
| 16 | RF-IVP | 6 | 4 | 6 | -48 | 26 | B-ExB | - |  |  |
| 18 | RF-IVP | 4 | 5 | 6 | -24 | 45 | B | - |  |  |
| 20 | RF-IVP | 1 | 4 | 6 | -48 | 29 | B | - |  |  |
| 21 | RF-IVP | 7 | 4 | 6 | -48 | 42 | ExB | + | 115 | 3 |
| 23 | RF-IVP | 7 | 5 | 6 | -24 | 45 | ExB | + | Abortion |  |
| 24 | RF-IVP | 5 | 4 | 6 | -48 | 59 | ExB-HB | + | 114 | 7 |
| M=Morulae, B=Blastocyst, ExB=Expanded Blastocyst, HB=Hatched Blastocyst | | | | | | | | | | |
